# Cerebral amyloid angiopathy-related inflammation: findings on magnetic resonance imaging

**DOI:** 10.1590/0100-3984.2017.0117

**Published:** 2019

**Authors:** Bruno Niemeyer de Freitas Ribeiro, Bernardo Carvalho Muniz, Edson Marchiori

**Affiliations:** 1 Instituto Estadual do Cérebro Paulo Niemeyer - Departamento de Radiologia, Rio de Janeiro, RJ, Brazil.; 2 Universidade Federal do Rio de Janeiro (UFRJ), Rio de Janeiro, RJ, Brazil.

**Figure 1:**
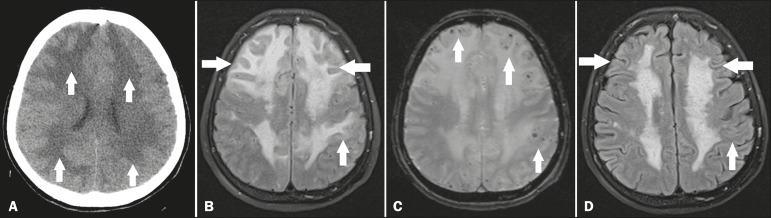
**A:** Noncontrast axial CT showing marked, diffuse bilateral hypodensity, predominantly in the white matter (arrows), making the cortical sulci and fissures less prominent. **B:** Axial FLAIR MRI sequence showing a diffuse bilateral hyperintense signal, predominantly in the white matter of the frontal lobes (arrows). **C:** T2-weighted gradient-echo MRI sequence showing multiple, dispersed foci with hypointense signals, most at the cortico-subcortical junction (arrows), suggestive of microhemorrhages. **D:** Axial FLAIR MRI sequence showing fewer foci of hyperintense signals after pulse therapy with methylprednisolone (arrows).

Dear Editor,

An 83-year-old female presented with a one-month history of daily non-pulsatile diffuse headaches that were refractory to analgesics, accompanied by discrete lower limb paresis. The patient also had systemic hypertension that was well controlled with medication. She reported no recent history of trauma, fever, or travel. A complete blood count showed no abnormalities, and the serology for HIV was negative, as was the VDRL test. Computed tomography (CT) of the skull showed diffuse hypodensity, predominantly in the white matter, making the sulci and fissures less prominent (Figure 1A). Magnetic resonance imaging (MRI) showed a hyperintense signal in T2-weighted and FLAIR sequences, without restricted diffusion, throughout the deep, periventricular white matter, predominantly in the frontal lobes, accompanied by multiple hypointense foci in a susceptibility-weighted imaging sequence, suggestive of microhemorrhages (Figures 1B and 1C). In view of those findings, the working diagnosis was cerebral amyloid angiopathy-related inflammation (CAA-ri), which was later confirmed by biopsy. Pulse therapy with methylprednisolone was initiated, resulting in an improvement in the symptoms and in the imaging findings by two weeks after the start of the treatment (Figure 1D).

Recent studies in the radiology literature of Brazil have emphasized the importance of MRI for improving central nervous system diagnoses^(^1^,^^2^^)^. CAA-ri is a rare disease that typically affects patients between 60 and 80 years of age, with no predilection for either gender, manifesting clinically as a subacute cognitive decline, headache, convulsion, focal neurological deficits, and neuropsychiatric disorders^(^3^-^7^)^. The pathophysiology of CAA-ri is not well known. However, it is known that it consists in the pathological accumulation of beta-amyloid in the media and adventitia of small and medium cortical and leptomeningeal vessels, accompanied by a perivascular lymphocytic inflammatory process, although it remains unknown which process occurs first^(^3^-^7^)^.

On CT, the classical presentation of CAA-ri is unifocal cortical and subcortical hypodensity, predominantly in the parietal lobes; although diffuse involvement can occur, it is less common and is usually asymmetric^(^3^-^7^)^. On MRI, hyperintense signals without restricted diffusion (characteristic of vasogenic edema) can be seen in T2-weighted and FLAIR sequences of the white matter, and susceptibility-weighted imaging sequences can show hypointense foci, due to microhemorrhages^(^3^-^7^)^. There can also be leptomeningeal enhancement adjacent to the areas of edema, superficial siderosis, and lobar infarction/hemorrhage, although those findings are more common in patients with non-inflammatory cerebral amyloid angiopathy^(^3^-^7^)^.

The differential diagnosis of multiple foci of microhemorrhage is broad and includes the following diagnoses^(^3^-^8^)^: non-inflammatory cerebral amyloid angiopathy, beta amyloid-associated angiopathy, diffuse axonal injury, poorly controlled arterial hypertension, thrombotic microangiopathy, sepsis, fat embolism, and malaria.

The treatment of CAA-ri consists of pulse therapy with methylprednisolone, with or without the use of immunosuppressive drugs, such as methotrexate, mycophenolate mofetil, and (most commonly) cyclophosphamide. However, nearly 60% of patients die or do not improve^(^3^-^7^)^.

In conclusion, although rare, CAA-ri should be considered in the differential diagnosis of multiple foci of microhemorrhage accompanied by edema, especially when clinical and laboratory findings exclude other diagnostic possibilities.
